# Lateral lymph node dissection for mid-to-low rectal cancer: is it safe and effective in a practice-based cohort?

**DOI:** 10.1186/s12893-021-01053-1

**Published:** 2021-01-21

**Authors:** Masakatsu Numata, Hiroshi Tamagawa, Keisuke Kazama, Shinnosuke Kawahara, Sho Sawazaki, Toru Aoyama, Yukio Maezawa, Kazuki Kano, Akio Higuchi, Teni Godai, Yusuke Saigusa, Hiroyuki Saeki, Norio Yukawa, Yasushi Rino

**Affiliations:** 1 Department of Surgery, Yokohama City University, 3-9 Fukuura, Kanazawa-ku, Yokohama, Kanagawa 236-0004 Japan; 2 Department of Surgery, Yokohama Minami Kyosai Hospital, 1-21-1 Mutsuurahigasi, Kanazawa-ku, Yokohama, Kanagawa 236-0037 Japan; 3 Department of Surgery, Fujisawa Shonandai Hospital, 2345 Takakura, Fujisawa, Kanagawa 252-0802 Japan; 4 Department of Biostatistics, Yokohama City University, 3-9 Fukuura, Kanazawa-ku, Yokohama, Kanagawa 236-0004 Japan

**Keywords:** Lateral lymph node dissection, Long-term, Practice-based cohort, Rectal cancer, Short-term

## Abstract

**Background:**

Most evidence regarding lateral lymph node dissection for rectal cancer is from expert settings. This study aimed to evaluate the safety and efficacy of this procedure in a practice-based cohort.

**Methods:**

A total of 383 patients who were diagnosed with stage II–III mid-to-low rectal cancer between 2010 and 2019 and underwent primary resection with curative intent at a general surgery unit were retrospectively reviewed. After propensity matching, 144 patients were divided into the following groups for short- and long-term outcome evaluation: mesorectal excision with lateral lymph node dissection (n = 72) and mesorectal excision (n = 72).

**Results:**

This practice-based cohort was characterized by a high pT4 (41.6%) and R1 resection (10.4%) rate. Although the operative time was longer in the lateral dissection group (349 min vs. 237 min, *p* < 0.001), postoperative complications (19.4% vs. 16.7%, *p* = 0.829), and hospital stay (18 days vs. 22 days, *p* = 0.059) did not significantly differ; 5-year relapse-free survival (62.5% vs. 66.4%, *p* = 0.378), and cumulative local recurrence (9.7% vs. 15.3%, p = 0.451) were also in the same range in both groups. In the seven locally recurrent cases in the lateral dissection group, four had undergone R1 resection.

**Conclusions:**

Lateral lymph node dissection was found to be safe in this practice-based cohort; however, the local control effect was not obvious. To maximize the potential merits of lateral lymph node dissection, strategies need to be urgently established to avoid R1 resection in clinical practice.

## Background

The incidence of lateral lymph node metastasis (LLNM) in stage II/III low rectal cancer is reported to be 7–20% [1–5]; it is associated with local recurrence and poor overall survival.

In Japan, lateral lymph node dissection (LLD) has been the main treatment strategy for LLNM [6]. Recently, the JCOG (Japanese Clinical Oncology Group) 0212 trial reported on the safety and efficacy of LLD [4, 5]. The results of this trial showed similar morbidity rates and relapse-free survival (RFS) between mesorectal excision (ME) followed by LLD (ME + LLD) and ME alone groups, with lesser local recurrence in the former; the investigators concluded that ME + LLD should be considered the standard surgical procedure for stage II/III low rectal cancer in Japan. However, while considering the application of LLD to clinical practice, two factors need to be considered. First, only surgeons from 33 Japanese institutions who specialized in LLD had participated in the JCOG0212 study. Therefore, the results of this technically demanding technique in practice-based settings may differ from those of the JCOG0212 trial. Second, the JCOG0212 trial excluded patients with clinical LLNM, which accounts for almost 20% of cases with locally advanced low rectal cancer [7]. Therefore, the impact of LLD on local control in mid-to-low rectal cancer remains unclear.

The aim of this study was to evaluate the safety and efficacy of LLD for mid- to low-rectal cancer in a practice-based cohort.

## Methods

### Study design

In this study, a practice-based cohort was defined as follows: (1) from a hospital without an independent colorectal surgery department, and (2) from a hospital, which was not an official member of the JCOG colorectal group. Among the nine group hospitals related to our department (Yokohama City University, Department of Surgery), only one was a specialized cancer center (Kanagawa Cancer Center Hospital, Kanagawa, Japan); it is an official member institute of the JCOG and has an independent colorectal surgery department; the other eight hospitals are not members of the JCOG and perform colorectal surgery in a general surgery department. Among these eight hospitals, three maintain a developed colorectal database. Therefore, this study used the databases of these three general surgery departments to obtain data for the practice-based cohort; the data included patient characteristics, preoperative assessments, operative characteristics, postoperative complications, pathological characteristics, and follow-up data.

**Fig. 1:**
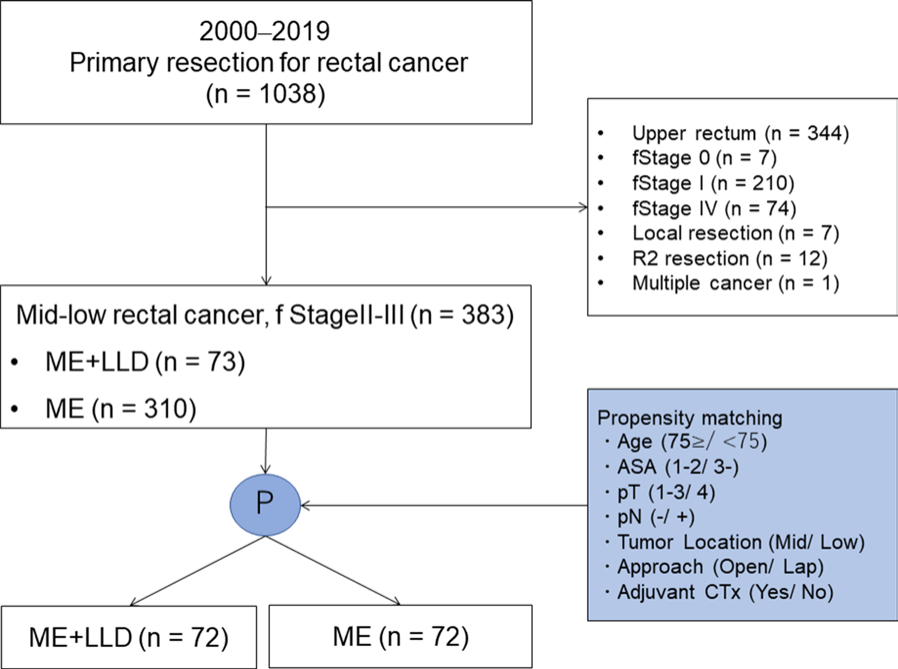
Patient flow diagram

Between April 2000 and March 2019, 1038 patients underwent primary resection for rectal cancer in this study cohort. Among these 1038 patients, 344 with upper-rectal cancer, 7 with stage 0 disease, 210 with stage I disease, 74 with stage IV disease, 7 with local resection, 12 with R2 resection, and 1 with multiple cancer resections were excluded. Thus, 383 patients with stage II–III mid-, to low-rectal cancer were included in the propensity-matching process. Finally, 144 matched patients were divided into the following groups: ME followed by LLD (ME + LLD) (n = 72) and ME alone (n = 72) (Fig. 1).

### Categorization of operator surgeons

Since technical skill may affect the outcomes of LLD, the operator surgeons were categorized into two groups as follows: trained surgeons for colorectal surgery (TS) and non-trained surgeons for colorectal surgery (NTS). As mentioned previously, our university has one cancer center hospital with a colorectal surgery unit. In our training system, surgeons from our university department usually work at any of the nine group hospitals, changing working hospitals every 2 years. In this study, trained surgeons were defined as those who had undergone training at the colorectal surgery unit of our cancer center hospital for 2 years, while those who had not, were defined as non-trained surgeons.

### Preoperative diagnosis

Preoperative evaluation included digital rectal examination, colonoscopy, histological examination, computed tomography (CT), magnetic resonance imaging, and barium enema. Patients were staged using the tumor node metastasis (TNM) staging system of the American Joint Committee on Cancer Staging Manual [8]. The TNM staging system considers LLNM as distant metastasis; however, the present study considered LLNM as regional metastasis.

### Indications for LLD and neoadjuvant chemoradiotherapy

According to the Japanese Society for Cancer of the Colon and Rectum (JSCCR) Guidelines for the Treatment of Colorectal Cancer [6], LLD is recommended when the lower border of the tumor is located in the lower rectum and has invaded beyond the muscularis propria (cT3–4). In this practice-based study, the actual indication was not standardized; it was ascertained at the discretion of the surgeon after considering tumor factors, patient characteristics, and surgeon preferences or experience.

In this study cohort, neoadjuvant chemoradiation therapy (neo-CRT) was only administered in cases with suspected resection margin positivity based on findings of preoperative imaging.

### Operative procedure and the extent of LLD

**Fig. 2:**
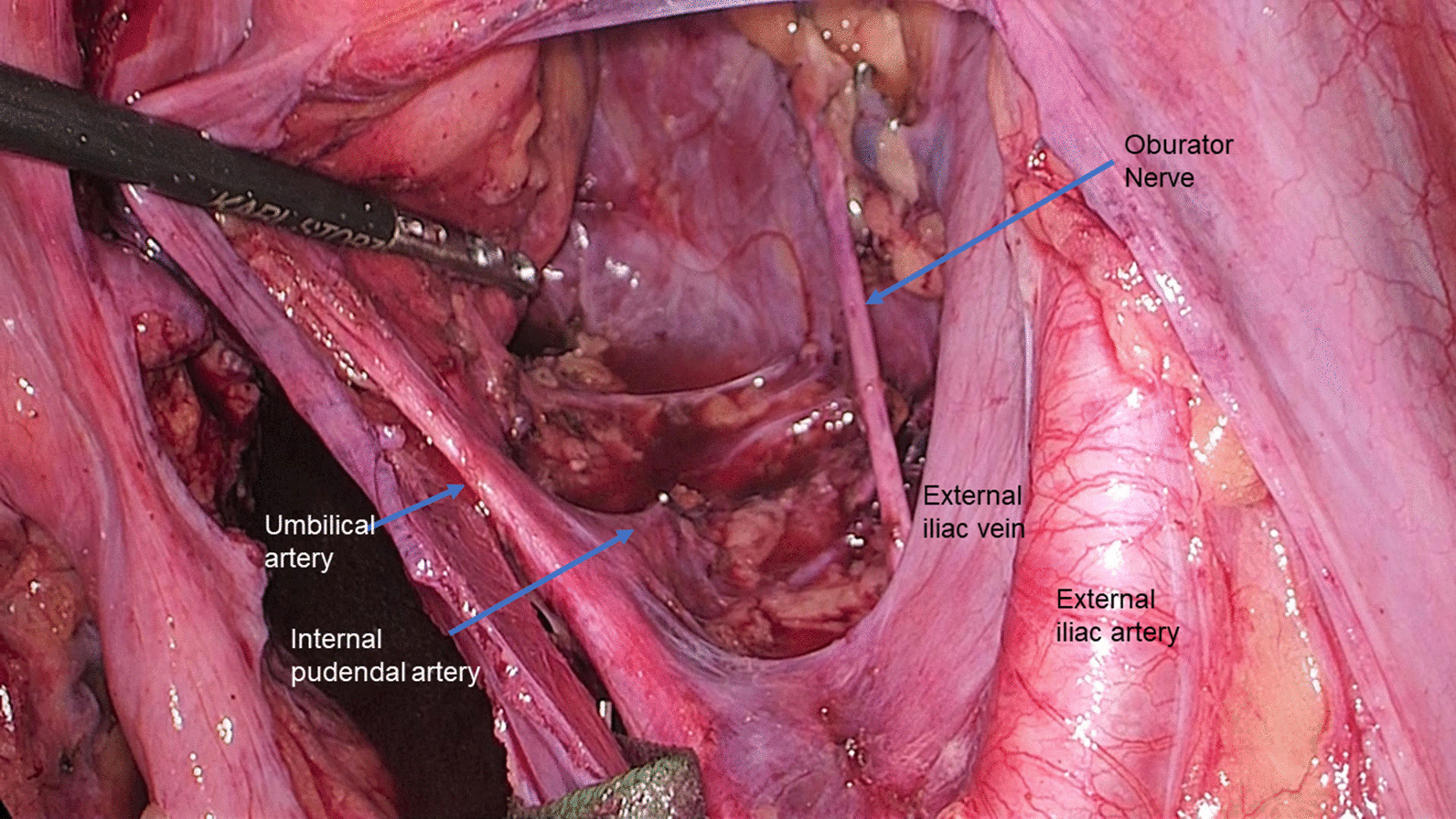
Image after right lateral lymph node dissection

The target lymph nodes in LLD are the internal iliac lymph nodes (ILNs) and obturator lymph nodes (OLNs). Bilateral dissection of these lymph nodes was performed after removal of the rectum (Fig. 2). The schematic image of the lateral lymph nodes and the details of the procedure have been described previously [9]. In the present study, the extent of LLD was categorized into either of the two patterns defined in the Japanese Classification of Colorectal, Appendiceal, and Anal Carcinoma [10]; in LD2 dissection, bilateral ILNs and OLNs were dissected, while LD1 dissection involved a less extensive area than LD2 dissection, i.e., unilateral dissection.

### Postoperative complications

Postoperative complications were evaluated using the Clavien–Dindo classification system [11]. In this study, grade 3–5 postoperative complications that occurred during hospitalization and/or within 30 days after surgery were recorded.

### Patient follow-up

Patients were followed up at outpatient clinics. Hematological tests, including serum carcinoembryonic antigen (CEA) and carbohydrate antigen 19-9 levels, physical examinations, and CT examinations were performed every 6 months for 5 years after surgery.

### Outcome of interest

The primary outcome of interest was the RFS rate, and the cumulative rate of local recurrence; the secondary outcome of interest was the postoperative complication rate.

### Propensity-score matching and statistical analyses

Propensity-score matching of patients who did and did not undergo LLD was performed based on their baseline characteristics. Patients in the ME alone group were matched in a 1:1 ratio to those in the ME + LLD group based on the following factors: age (75≥/<75), American Society of Anesthesiologists (ASA) classification (1–2/3–5), pT (1–3/4), pN (negative/positive), tumor location (mid rectum/low rectum), approach (open/laparoscopic), and adjuvant chemotherapy use (yes/no). The standardized difference for all matching factors were confirmed to be less than 0.25. The significance of association between the study groups and clinicopathological parameters was assessed using the Fisher’s exact or *t*-tests. The RFS curves were constructed using the Kaplan–Meier method and compared using the log-rank test.

All statistical analyses were performed using EZR [12] (Jichi Medical University, Saitama, Japan) and R software (version 3.4.3). All *p*-values were two-sided, and a *p*-value < 0.05 was considered to indicate statistical significance.

## Results

### Patient characteristics

The baseline characteristics of the ME + LLD and ME alone groups are presented in Table 1. There were no significant differences between the two groups with regard to age, body mass index, ASA classification, preoperative serum CEA levels, tumor location, pT, pN, pStage, neo-CRT, histopathological type, tumor diameter, and adjuvant chemotherapy. With regard to sex, the ratio of male patients was significantly higher in the ME + LLD group than in the ME alone group. In the pre-matching cohort, the proportion of male patients in the LLD group is high; we could not find a plausible explanation for this observation. In the propensity matching process, we decided not to include the sex as a matching factor, because if included, the number of matched patients would be considerably low.

**Table 1:** Baseline characteristics (n = 144)

Parameter	ME + LLD (n = 72)	ME (n = 72)	*p*-value
Age	66 (36–83)	67.5 (34–90)	0.246
Sex (male/female)			< 0.001
Male	50 (69.4%)	21 (29.1%)	
Female	22 (30.6%)	51 (70.9%)	
Body mass index	23.0 (16.5–34.0)	22.3 (16.6–32.7)	0.334
ASA			0.244
1–2	63 (87.5%)	68 (94.4%)	
3–4	9 (12.5%)	4 (5.6%)	
Serum CEA (ng/ml)	4.3 (0.5–159.8)	3.6 (0.5–65.8)	0.132
Location			0.557
Mid rectum	15 (20.8%)	19 (26.4%)	
Low rectum	57 (79.2%)	53 (73.6%)	
pT			0.237
1–3	38 (52.7%)	46 (63.8%)	
4	34 (47.3%)	26 (36.2%)	
pN			1.000
Negative	30 (41.6%)	29 (40.2%)	
Positive	42 (58.4%)	43 (59.8%)	
pStage			1.000
II	30 (41.6%)	29 (40.2%)	
III	42 (58.4%)	43 (59.8%)	
pLLNM	10 (13.8%)	–	–
Neo-CRT	5 (6.9%)	0	0.058
Histological type			1.000
Tubular adenocarcinoma	67 (93.0%)	66 (91.6%)	
Other histological type	5 (7.0%)	6 (8.4%)	
Tumor diameter (mm)	50.0 (15–120)	45.0 (3–95)	0.293
Adjuvant CTx	35 (48.6%)	31 (43.0%)	0.616

Continuous variables are presented as medians with ranges. Discrete variables are presented as numbers and percentages

*ASA* American Society of Anesthesiologists, *CEA* carcinoembryonic antigen, *Mid* mid rectum, *Low* low rectum, *LLNM* lateral lymph node metastasis, *CRT* chemoradiation therapy, *CTx* chemotherapy

The five cases in the ME + LLD group underwent preoperative CRT, compared to none in the ME alone group (p = 0.058). The positive pathological LLN (pLLN) was detected in 10 cases (13.8%) of the ME + LLD cohort. The median follow-up period of the entire cohort was 36.7 months.

### Operating surgeons

**Fig. 3:**
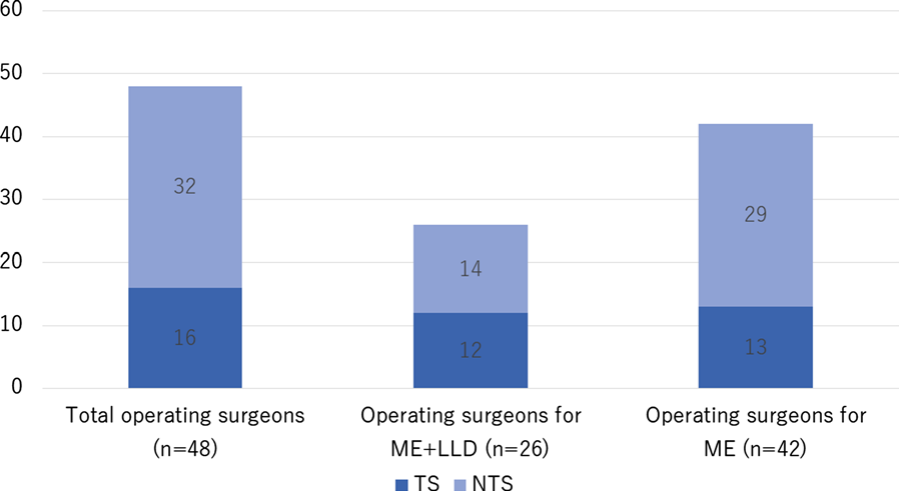
Number of operating surgeons. *ME* mesorectal excision, *LLD* lateral lymph node dissection, *TS* trained surgeon for colorectal surgery, *NTS* non trained surgeon for colorectal surgery

The numbers of operating surgeons are presented in Fig. 3. The total number of operating surgeons for this cohort was 48, of which 16 and 32 were TS and NTS, respectively. In the ME + LLD group, the total number of operating surgeons was 26; among them, 12 (46.1%) were TS. In the ME alone group, the total number was 42, of whom 13 (30.9%) were TS.

**Fig. 4:**
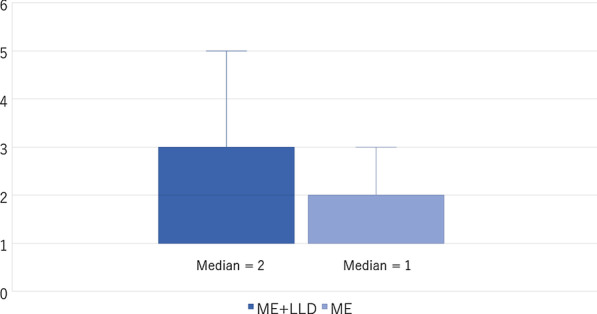
Number of operations by each operating surgeon. *ME* mesorectal Excision, *LLD* lateral lymph node dissection

The numbers of operations performed by each surgeon are presented in Fig. 4. The median numbers of the ME + LLD and ME alone surgeons were 2 and 1, respectively.

These findings suggest that the data includes results from various surgeons, a not those from a limited number of well-trained experts.

### Short-term outcomes

The short-term outcomes of the patients are summarized in Table 2. With regard to the extent of LLD in the ME + LLD group, the rate of LD2 and LD1 were 84.7% and 15.3%, respectively. In the ME + LLD group, 37 (51.3%) operations were directly performed by TS, compared to 28 (38.9%) in the ME alone group (*p* = 0.180). The operative time was longer in the ME + LLD group than in ME alone group (349 min vs. 237 min, *p* < 0.001). With regard to postoperative complications, there was no marked difference between the ME + LLD and ME alone groups (19.4% vs. 16.7%, *p* = 0.829), and each complication was similar between both groups; no mortality was noted. The postoperative hospital stay did not significantly differ between the groups (18 days vs. 22 days, *p* = 0.059). The R1 resection rate for the ME + LLD and ME alone groups were 9.7%, and 11.1%, respectively (*p* = 1.000). Among 15 cases with R1 resection, 13 underwent adjuvant chemotherapy, and two underwent observation without adjuvant therapy. The numbers of harvested lymph nodes were significantly higher in the ME + LLD group than in ME alone group (31 vs. 16, *p* < 0.001).

**Table 2:** Short-term outcomes (n = 144)

Parameter	ME + LLD (n = 72)	ME (n = 72)	*p*-value
Approach			0.300
Open	42 (58.3%)	49 (68.0%)	
Laparoscopic	30 (41.7%)	23 (32.0%)	
Sphincter preserved	37 (51.3%)	39 (54.1%)	0.868
Extent of LLD			
LD2	61 (84.7%)	–	
LD1	11 (15.3%)	–	
Operated by			0.180
TS	37 (51.3%)	28 (38.8%)	
NTS	35 (48.7%)	44 (61.2%)	
Operative time (min)	349 (110–834)	237 (110–623)	< 0.001
Blood loss (gram)	392 (10–2770)	295 (5–9892)	0.270
Complications (CD ≥ Grade 3)	14 (19.4%)	12 (16.7%)	0.829
Anastomotic leakage	4 (5.6%)	6 (8.3%)	0.745
Ileus	4 (5.6%)	3 (4.2%)	1.000
Abdominal abscess	2 (2.8%)	2 (2.8%)	1.000
Pulmonary embolism	1 (1.4%)	0 (0.0%)	1.000
Wound dehiscence	1 (1.4%)	0 (0.0%)	1.000
Stoma perforation	1 (1.4%)	0 (0.0%)	1.000
Abdominal abscess	1 (1.4%)	0 (0.0%)	1.000
Respiratory suppression due to anesthetic overdose	0 (0.0%)	1 (1.4%)	1.000
Mortality	0 (0.0%)	0 (0.0%)	–
POS (days)	18 (8–114)	22 (8–72)	0.059
R1 resection	7 (9.7%)	8 (11.1%)	1.000
Harvested lymph node	31 (10–109)	16 (2–83)	< 0.001

Continuous variables are presented as medians with ranges. Discrete variables are presented as numbers with percentages

*LLD* Lateral lymph node dissection, *TS* Trained surgeon for colorectal surgery, *NTS* Non-trained surgeon for colorectal surgery, *CD* Clavien–Dindo classification, *POS* Postoperative hospital stay

^a^Other complications include pulmonary embolism (n = 1), wound dehiscence (n = 1)

### Survival outcome

**Fig. 5:**
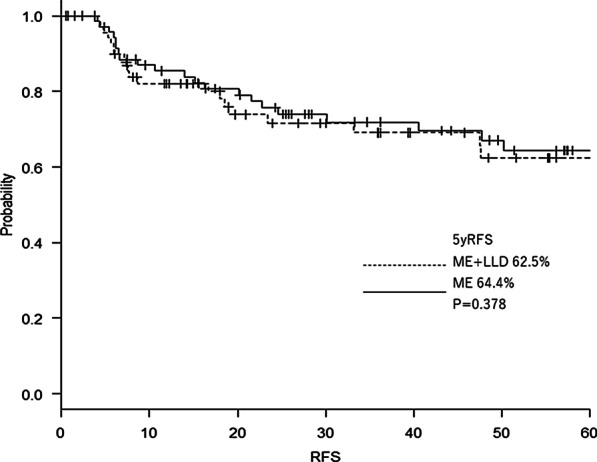
Relapse-free survival in the ME + LLD and ME groups. *ME* mesorectal excision, *LLD* lateral lymph node dissection, *RFS* relapse-free survival

RFS analysis showed similar curves in the ME + LLD and ME alone groups, with non significant difference in the 5-year RFS rate between the groups (62.5% vs. 66.4%, *p* = 0.378) (Fig. 5).

### Recurrence pattern

The proportion of patients with any recurrence in the follow-up period was similar between the ME + LLD and ME alone groups (27.8% vs. 26.4%, *p* = 1.00, respectively) (Table 3). In both groups, local recurrence was the most common pattern of relapse (9.7% vs. 15.3%, *p* = 0.451). There was no significant difference in the recurrence pattern between the groups.

**Table 3:** Recurrence pattern (n = 144)

	ME + LLD (n = 72)	ME (n = 72)	*p*-value
All recurrence	20 (27.8%)	19 (26.4%)	1.000
Local	7 (9.7%)	11 (15.3%)	0.451
Liver	6 (8.3%)	3 (4.2%)	0.494
Lung	2 (2.8%)	5 (6.9%)	0.441
Peritoneal dissemination	2 (2.8%)	1 (1.4%)	1.000
Distant lymph node	6 (8.3%)	2 (2.8%)	0.441
Others	1 (1.4%)	1 (1.4%)	1.000

## Discussion

In the JSCCR guidelines [6], ME + LLD is recommended as a standard procedure for locally advanced lower rectal cancer on the basis of its local control effect demonstrated in the JCOG0212 trial [4, 5]. However, most of the reported evidence on ME + LLD is from a limited number of surgeons specializing in this procedure [3–5, 13–15]; practice-based evidence is lacking. Recently, Sakai et al. reported that ME + LLD was performed in 30% of locally advanced low rectal cancer cases in real-world Japanese practice [16]; this indicates a discrepancy between guideline recommendations and on-site practice.

The outcomes of ME + LLD in practice-based settings need to be confirmed, and the problems in this setting need to be identified. This study confirmed that ME + LLD did not increase the incidence of postoperative complications; however, it should be noted that ME + LLD demonstrated no beneficial effect on local recurrence in this cohort.

First, regarding safety, the median operative time was 112 min longer in ME + LLD than ME alone (349 vs 237 min); the corresponding time in the JCOG0212 cohort was 106 min. The consistency of these results indicates that the additional time for LLD amounts to approximately 110 min in both practice-based and specialized facilities.

Regarding the postoperative complications, their incidence in the ME + LLD group was similar to that in the ME alone group (19.4% vs. 16.7%, *p* = 0.829); the corresponding incidence in the JCOG0212 was 22%, which agreed with our data.

In addition, it is worth noting that the present study reflects the results from 48 various surgeons, and the median number of operations per each surgeon was very low.

In general, LLD is recognized as a challenging procedure owing to the complex pelvic anatomy, which potentially leads to postoperative complications [17]. Therefore, the present data needs to be interpreted with caution. In the ME + LLD group, 51.3% of total operations were directly performed by TS, and the proportion of operations where TS directly operated or participated as the leading assistant was 93% (67 cases) (data not shown in the table). Most ME + LLD operations were directly performed or supervised by TS; this may have contributed to the safety of the ME + LLD group. Moreover, when considering the established concept that there is a presence of a volume-outcome relationship in a complex colorectal cancer surgery [18], there was a possibility that operation performed by TS was somewhat superior regarding morbidity than those by NTS, which was not analyzed in this study.

Regarding long-term results, this study confirmed that as demonstrated by previous studies, LLD does not improve RFS [5, 19]. In the present study, the 5-year RFS for ME + LLD and ME alone were 62.5% and 64.4 respectively; these were worse than those of JCOG0212, at 73.4% and 73.3% respectively. This discrepancy may be attributed to the difference in the tumor characteristics between the studies. The rate of pLLN (+) and pT4 in the ME + LLD group of the JCOG0212 were 7.4% and 3.1%, respectively, whereas those in this study were 13.8% and 47.3%, respectively.

In the median follow-up period of 36.7 months in this cohort, local recurrence was observed in 9.7% and 15.3% of cases the ME + LLD and ME alone groups, respectively, and there was no significant reduction in the local recurrence rate in the ME + LLD group (p = 0.451). Conversely, in the JCOG0212 study, the local recurrence rates in the ME + LLD and ME alone groups were 7.4% and 12.5%, respectively, in the median follow-up period of 72.2 months, with significant difference (*p* = 0.02). There are three probable explanations for the lack of decline in the local recurrence rate in the ME + LLD group in this study. First, as mentioned above, this study included many cases with more locally advanced disease; consequently, the R1 resection rate in the entire cohort was 10.4%. Among the seven cases with local recurrence in the ME + LLD group, four had undergone R1 resection. Therefore, the local control effect of LLD may have been undermined by the R1 resection. Oki et al. also investigated the effect of LLD on lower rectal cancer in a subset analysis of a clinical trial, that validated the benefits of adjuvant chemotherapy in rectal cancer [19]. The 3-year local recurrence rate in the ME + LLD and ME alone groups were 16.6% and 15.4%, respectively, without any significant difference. Although the rate of R1 resection was not reported in the study, the higher proportion of pT4 in the LLD group (8.4%) may have negatively affected local control in the LLD group.

The second factor was the extent of LLD. In the JCOG0212, systematic LD2 dissection was performed in all cases as per the study protocol, whereas in this cohort, the rate of LD2 was 84.7%. Therefore, limited dissection (LD1) was performed in 15.3% of cases, which may have led to a decrease in local control. Indeed, Kanemitsu et al. have shown that unilateral LLN dissection is a significant risk factor for local recurrence [13].

The third factor was the lack of power due to the small sample size and short follow-up period.

In summary, this study showed that the complications and RFS in this practice-based cohort were not widely different from those of cohorts treated by experts. However, a significant local control effect of LLD was not observed in the present study, possibly due to local recurrence following R1 resection, and the occasional omission of systematic LLD. The present results suggest the need for establishing strategies to avoid R1 resection in clinical practice.

The limitations of the present study should be considered when interpreting the results. First, there may have been selection bias when considering patients for LLD. Propensity-score matching was performed to eliminate selection bias as far as practicable and to balance the cohort; however, due to reasons mentioned previously, sex remained a significantly different factor. Besides, neither operating surgeons nor neo-CRT were included as matching factor, which might influence on clinical outcomes. The second is that in this study, TS performed most cases in the ME + LLD group; it should be recognized that they have anatomical knowledge and surgical experience of the lateral region. The third is that the present database lacked information regarding the precise location of local recurrences, namely, anastomotic, central, and lateral. Therefore, it was impossible to ascertain whether LLD reduced lateral recurrence in this cohort. The fourth is that the follow-up period was insufficient in part of patient to draw the 5-year survival rate. Despite these limitations, we believe that our findings will improve the understanding of the current situation and the issues associated with LLD in clinical practice.

## Conclusions

The short-term outcomes and RFS following ME + LLD in the practice-based cohort were similar to that of the expert setting. However, adequate local control could not be confirmed in this cohort, probably due to the relatively high rate of R1 resection and the occasional omission of systematic LLD. Adequate preoperative assessment for resection margin, solid skill to secure surgical margin, and appropriate judgement for neoadjuvant chemoradiotherapy under margin threatening condition are essential for further improvement in locally advanced rectal cancer.

## Data Availability

The datasets used during the current study are available from the corresponding author on reasonable request. The authors did not used any publicly available data in this study.

## Abbreviations

LLNM: Lateral lymph node metastasis
LLD: Lateral lymph node dissection
JCOG: Japanese Clinical Oncology Group
RFS: Relapse-free survival
ME: Mesorectal incision
TS: Trained surgeons for colorectal surgery
NTS: Non-trained surgeons for colorectal surgery
TNM: Tumor node metastasis
JSCCR: Japanese Society for Cancer of the Colon and Rectum
neo-CRT: Neoadjuvant chemoradiotherapy
pLLN: Positive pathological LLN
CEA: Carcinoembryonic antigen
CT: Computed tomography
